# Long-Read Sequencing Outperforms Short-Read Sequencing in Detecting Most Structural Variations

**DOI:** 10.17161/sjm.v2i2.23671

**Published:** 2025-02-03

**Authors:** Xinyue Chen, Xiaodong Lu, Xianglin Shi, Shaojun Yu, Jonathan Zhao

**Affiliations:** 1 Department of Human Genetics, Emory University School of Medicine, Atlanta, GA 30322, USA;; 2 Department of Urology, Emory University School of Medicine, Atlanta, GA 30322, USA;; 3 Winship Cancer Institute, Emory University School of Medicine, Atlanta, GA 30322, USA;

**Keywords:** structural variation, long-read sequencing, short-read sequencing, whole-genome sequencing

## Abstract

Structural variations (SV) are common in the cancer genome and play critical roles in regulating tumorigenesis. In the past decades, many SVs have been detected through analyses of whole-genome sequencing (WGS) data generated mainly by Illumina paired-end short-read sequencing (SRS). Recent advances in long-read sequencing (LRS) techniques provide exciting opportunities for SV detection. However, a comprehensive analysis of the pros and cons of LRS and SRS in detecting SVs in a cancer genome is still lacking. Here, we performed WGS of the LNCaP prostate cancer cell line through LRS using the Oxford Nanopore Technology and called main SVs, which were compared to those derived from publicly available LNCaP SRS data. Strikingly, LRS is superior in detecting insertions of all sizes and deletions of <1000 bp long, whereas SRS is very useful in capturing long deletions, taking advantage of its paired-end reads. LRS identified more precise breakpoints of detected SVs. In addition, we found that SRS called many duplications and inversions, most of which were not confirmed by LRS, likely due to ambiguity in SRS read alignment to repetitive regions, leading to errors in SV calling. In conclusion, LRS outperformed SRS in detecting most SVs, except deletions longer than LRS read lengths. Our study highlights the advantages of LRS in resolving complex genomic rearrangements and underscores its potential for improving SV detection in cancer genomics.

## Introduction

Structural variations (SVs) represent a major class of genomic alterations in cancer, encompassing deletions, duplications, inversions, and insertions. These alterations play a critical role in tumorigenesis and disease progression, as seen in many cancers such as prostate cancer. Notably, SVs leading to the fusion of TMPRSS2 and ERG have been observed in approximately 50% of prostate cancer cases [1], while amplification of an enhancer upstream of the androgen receptor (AR) has been implicated in metastatic prostate cancer progression [2].

Previous studies have investigated SVs using whole-genome sequencing data (WGS) obtained primarily through paired-end short-read sequencing (SRS) by Illumina [3, 4]. However, SRS has intrinsic limitations in SV detection due to its short-read (typically ~100 bp for each read) nature but also has advantages in detecting large structural variations by producing paired-end reads [5]. On the contrary, long-read sequencing (LRS) produces reads often with an average length longer than 10 kb and substantially improves read alignment within repetitive genomic regions, which frequently mediate SV formation [6].

Few recent studies have started to compare SRS and LRS for SV detection [7, 8]. While SRS has been extensively optimized, with well-established SV calling algorithms capable of detecting a substantial pro-portion of SVs [5], LRS excels at resolving complex SVs, particularly those in highly repetitive regions. Additionally, LRS is highly effective in detecting long insertions, a category of SVs that is particularly challenging to reconstruct using short-read data. However, a comprehensive comparison of LRS and SRS in detecting SVs in prostate cancer has not been previously attempted.

Previous studies have characterized prostate cancer cell lines using SRS-based whole-genome and whole-exome sequencing [3, 4]. These efforts have successfully identified single nucleotide variations (SNVs), copy number variations (CNVs), SVs, and gene fusions. To systematically investigate the differences in SV detection between SRS and LRS in the LNCaP prostate cancer cell line, we performed long-read whole-genome sequencing using Oxford Nanopore Technology (ONT). We identified SVs from LRS data and compared the detection of different SV types with those derived from publicly available LNCaP SRS data. LRS proved superior in detecting insertions of all sizes and small deletions (<1000 bp), while SRS was more effective for long deletions due to its paired-end reads. Additionally, LRS provided more precise SV breakpoints, whereas SRS identified numerous duplications and inversions, many of which were not validated by LRS, likely due to alignment errors in repetitive regions. Overall, LRS outperformed SRS in detecting most SVs, except for deletions exceeding LRS read lengths. Our findings highlight the advantages of LRS in resolving complex genomic rearrangements and its potential for enhancing SV detection in cancer genomics.

## Results

### Pros and cons of LRS and SRS in detecting different types of SVs

**Figure 1.**
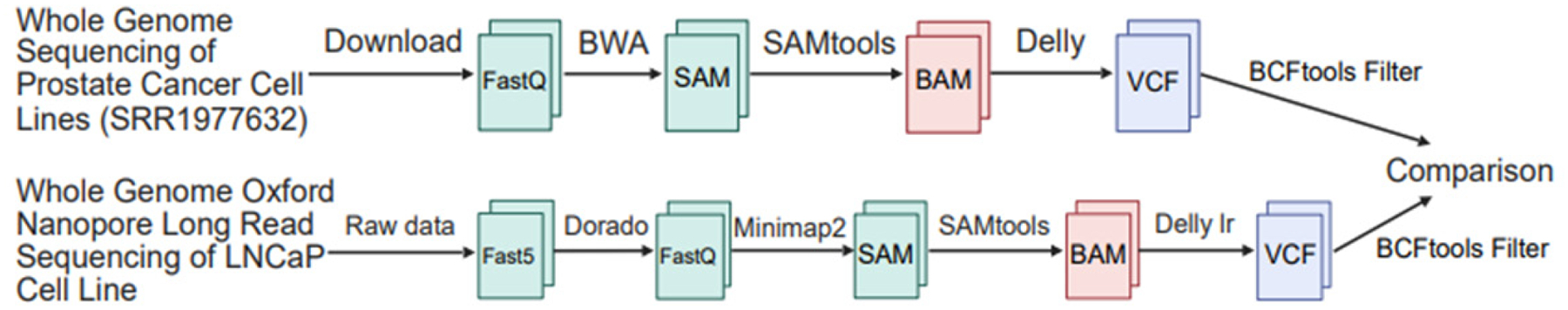
Workflow of data processing for structural variation comparison. Workflow of data analysis for SV detection and downstream comparison in LRS and SRS. Tools used (on arrows) and format changes (in rectangles) are shown for SRS (top panel) and LRS (bottom panel).

To obtain WGS data of LNCaP PCa cell lines, we performed LRS using the PromethION 2 Solo ONT sequencer. We obtained 58,245,198 LRS reads with average read lengths of 3,736 bp, leading to an overall 32x coverage of the human genome (Table 1). The raw LRS data in FAST5 format was base-called using Dorado [9], and aligned to the reference genome using Minimap2 [10] to create SAM files, which were converted to BAM files for variant calls (Fig 1). For comparison with SV calling with SRS, we obtained publicly available WGS data of the LNCaP cell line (SRR1977632) from the NCBI Short Read Archive, which includes 2,517,372,955 SRS, with average read lengths of 90 bp, leading to 63x genome coverage. SVs were called using delly [11] and delly long-read modules [11] for SRS and LRS, respectively, and the results were filtered by BCFtools [12] (Fig 1).

We next compared SV calling results from the LRS and SRS of LNCaP data. Interestingly, we found that LRS detected overall many more deletions and insertions, while SRS identified a lot more duplications and inversions (Table 2). Specifically, SRS data revealed 3,557 deletions (66.4%), 956 duplications (17.6%), 855 inversions (16.0%), and no insertions after filtering, whereas LRS data detected 9,072 deletions (35.7%), 99 duplications (0.4%), 96 inversions (0.4%), and 16,114 insertions (63.5%). Out of these, 2,178 deletions (61.2% of SRS-detected and 24.0% of LRS-detected deletions), 28 duplications (3.0% of SRS-detected and 28.3% of LRS-detected duplications), and 49 inversions (5.7% of SRS-detected and 51.0% of LRS-detected inversions) overlapped (Table 2). Therefore, while LRS and SRS detected substantially overlapping SVs, they each have pros and cons in identifying specific types of SVs, such as insertions, which LRS did well but SRS failed to detect.

### LRS, but not SRS, detects insertions

**Figure 2.**
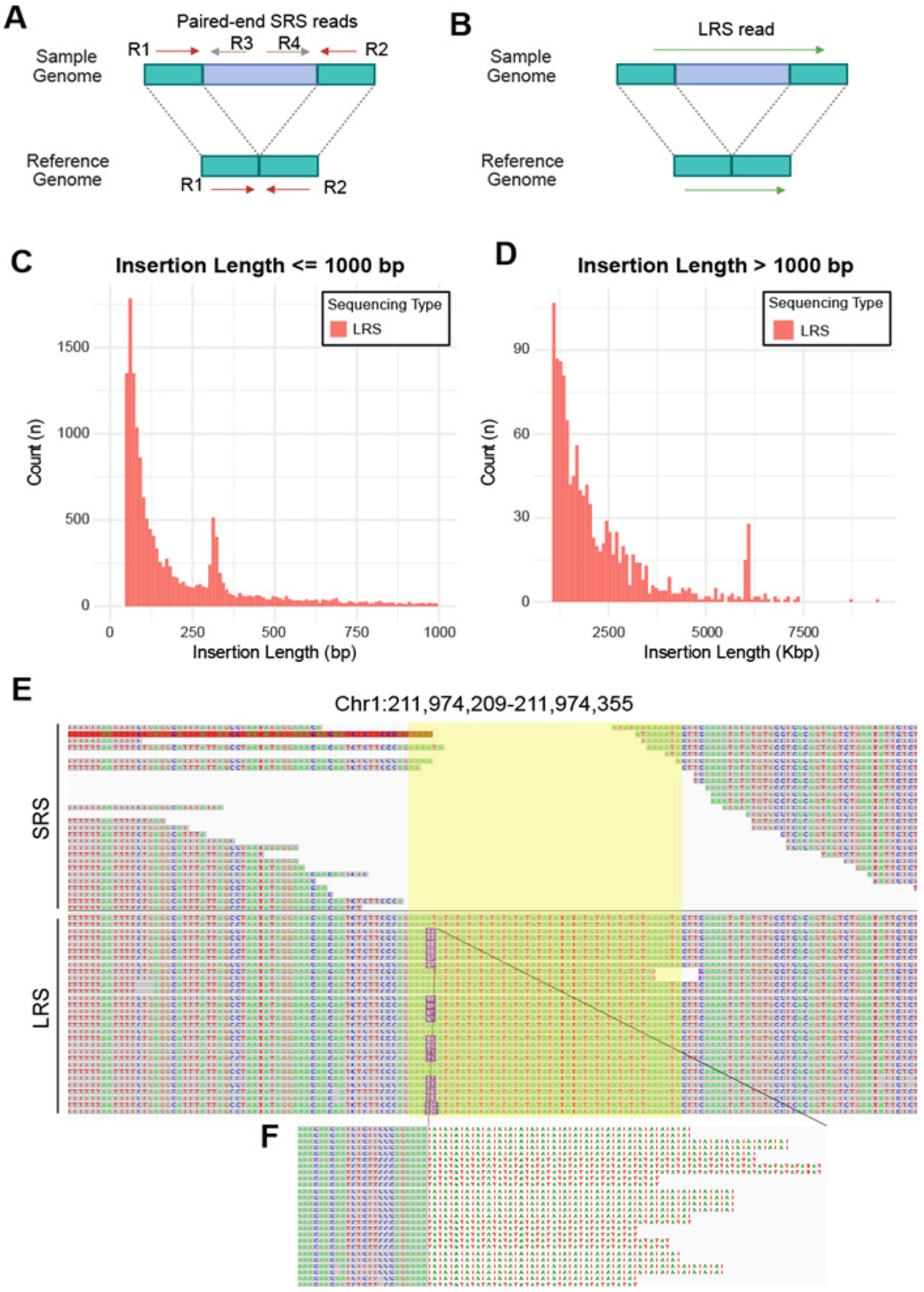
LRS detected many insertions, which SRS failed to capture. **A-B.** Approaches for detecting insertions by SRS and LRS. SRS (**A**) relies on paired-end reads to infer the type, size, and location of the DNA fragment. R1 and R2 from the sample genome (top) that contains an insertion (blue) could be mapped to the reference genome but won’t reveal an insertion. R3 and R4 will be discarded due to alignment failure. LRS (**B**) utilizes the alignment patterns of long reads to directly identify insertion. **C-D.** Stacked histograms displaying the number (count) of insertions detected by LRS (red) that were ≤ 1000 bp (n= 14872, **C**) or > 1000 bp (n= 1242; **D**). Note: much smaller Y-axis scale for D than C. **E-F.** An example of an insertion (purple box, zoomed in in F) called by LRS (bottom) that contains AT repeats (yellow highlighted region), where SRS (top) reads failed to align.

SRS has inherent limitations in detecting insertions because SRS reads from inserted sequences are unable to align to the reference genome and thus discarded, whereas read pairs flanking an insertion, albeit mappable to the reference genome, won’t reveal an insertion in between (Fig 2A). In contrast, the two ends of LRS flanking stretches of inserted sequences could be mapped to the reference genome and also enable the detection and retrieval of the exact inserted sequence (Fig 2B). This explains our finding that SRS and LRS detected 0 and 16,144 insertions, respectively (Table 2). Interestingly, we found that most (92%) of the LRS-detected insertions were less than 1 kb, with only a few exceeding 5 kb (Fig 2C–D, with different scales of Y-axes). This is due to the limitations that the average read length of our sample was 3,736 bp and that LRS provides single-end reads, thus unable to reveal SVs beyond the read length. Additionally, many of these insertions were at repetitive regions of the reference genome, where SRS reads often fail to align accurately. For instance, an insertion identified by LRS overlapped a highly repetitive region on chromosome 1, where no SRS reads were mapped, and no insertions were detected by SRS (Fig 2E). These findings highlight the advantage of LRS in detecting insertions, including those at repetitive regions, while SRS is intrinsically unable to capture insertions.

### LRS captures short deletions with high sensitivity and precision, while SRS excels in detecting very long deletions

**Figure 3.**
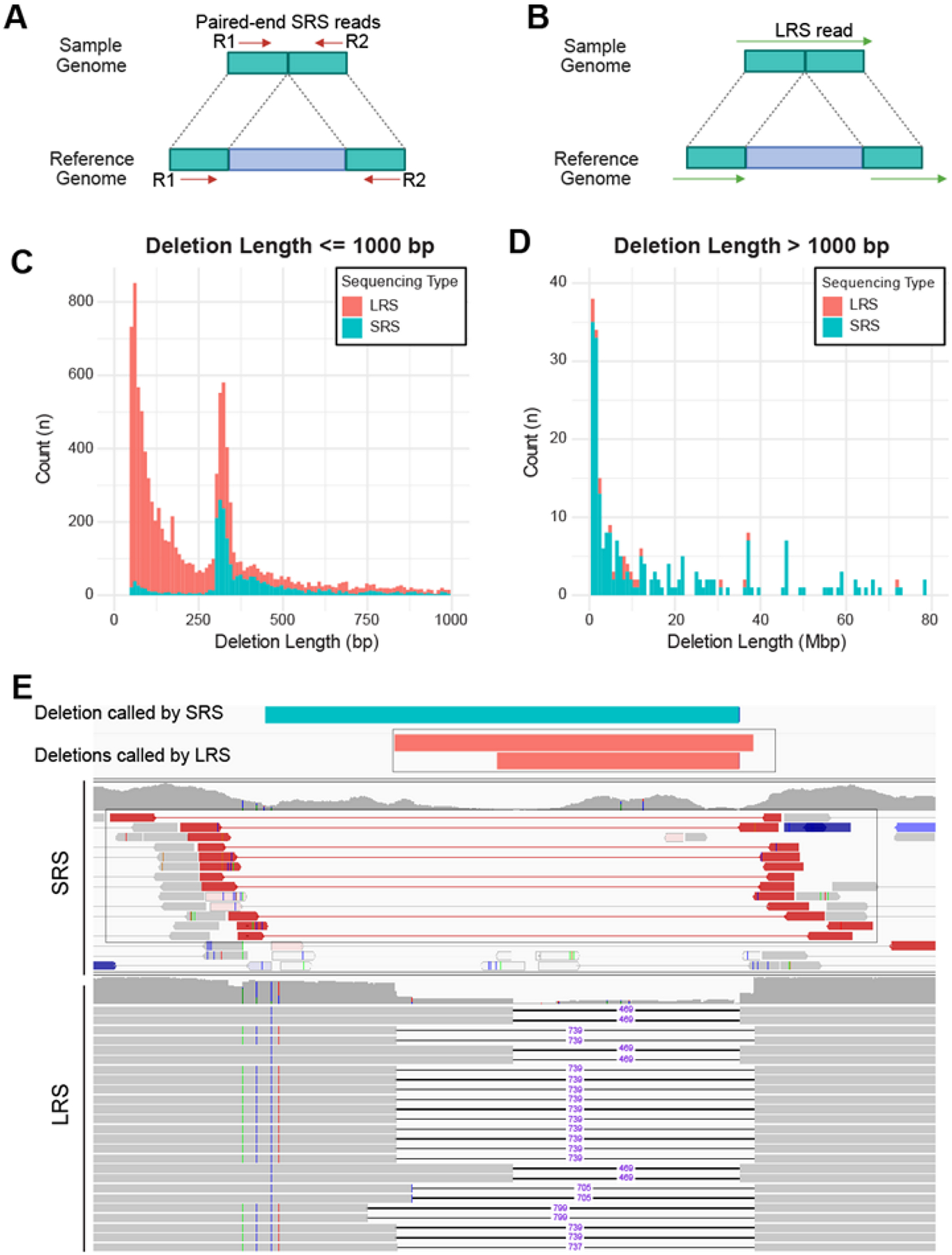
LRS calls short deletions with high sensitivity and precision, while SRS excels in detecting very long deletions. **A-B.** Approaches for detecting deletion by SRS and LRS. SRS (**A**) relies on paired-end reads to infer the type, size, and location of deletions. In contrast, LRS (**B**) utilizes the alignment patterns of long reads to directly identify deletions. **C-D.** Stacked histograms display the number of deletions detected by LRS (red) and SRS (blue). **C:** deletions ≤ 1000 bp and **D:** deletions > 1000 bp. The x-axis represents deletion length, and the y-axis indicates the count (n) of deletions. **E.** An example of a deletion called using SRS (top blue bar) and two deletions called using LRS (the two red bars) in the same genomic region. Shown underneath are the paired-end SRS reads (top) and single-end LRS reads (bottom). Black lines annotated by purple numbers indicate the number of deleted base pairs.

SRS calls deletion when the distance between the read pair alignment is much longer than the median distance of all paired alignments (Fig 3A), whereas LRS detects deletion when the two ends of a read are mapped to two discontinuous regions on the genome (Fig 3B). Surprisingly, we found that SRS missed deletions that were less than 300bp, which were successfully detected by LRS (Fig 3C). This limitation of SRS may be related to how the SV-calling software defines the cut-off distance to call a deletion. Interestingly, SRS was as effective as LRS in capturing deletions between 300 to 1000 bp long. Of note, SRS surpassed LRS in detecting very long deletions, due to such deletions being longer than the length of LRS reads and the limitation of LRS being a single-end sequencing technology (Fig 3D). Moreover, we found that LRS-based SV calling identified more deletions and defined the breakpoints more precisely in complex chromosome regions, such as telomeres and repetitive regions, where SRS often has challenges with sequence alignment. For instance, Figure 3E shows one region where paired-end SRS suggested a single deletion, while LRS resolved two distinct deletions of different lengths within the same region with very precise breakpoints (Fig 3E). These results indicate that LRS provides greater sensitivity and precision in the detection of short deletions, including those in complex genomic regions, where SRS excels in capturing very long deletions due to its paired-end nature.

### SRS calls many more duplications than LRS

**Figure 4.**
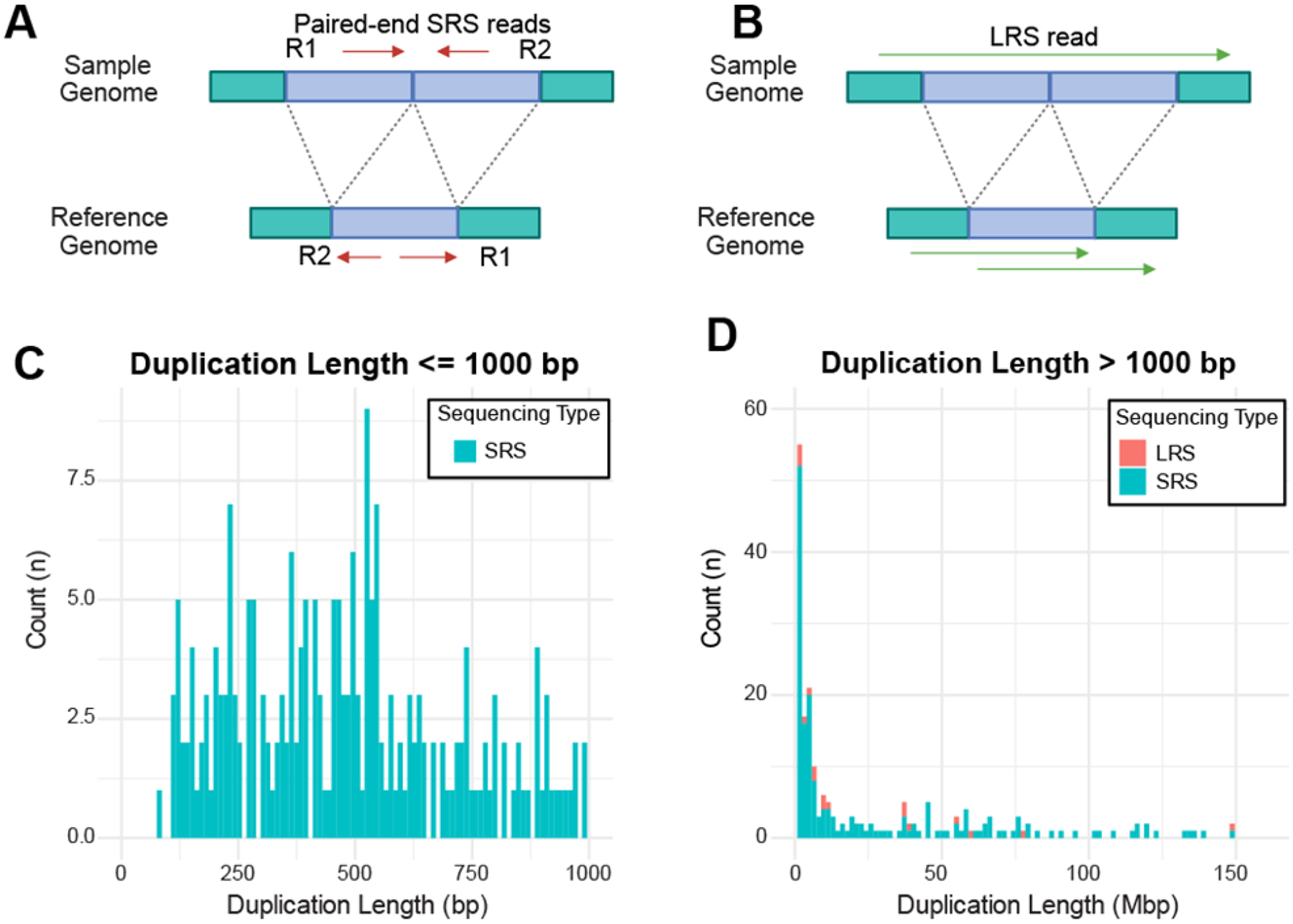
SRS captures many more duplications than LRS. **A-B.** Approaches for duplication identification by SRS and LRS. **C-D.** Stacked histograms displaying the number of duplications detected by LRS (red) and SRS (blue), with duplications ≤ 1000 bp (LRS: n=0; SRS: n=210, **C**) and duplications > 1000 bp (LRS: n=98; SRS: n=729, **D**). The x-axis represents duplication length, and the y-axis indicates the count (n) of duplications.

SRS calls duplications when a pair of SRS reads forms divergent, rather than converging pairs when aligned to the reference genome (Fig 4A). By contrast, LRS calls a duplication if two or more regions of one read maps to the same region on the reference genome (Fig 4B). Interestingly, we found that SRS identified many short duplications of less than 1,000bp, but LRS detected no duplications in this size range (Fig 4C). SRS also called many long duplications of 1000bp to 150Mbp, while LRS only captured a few (Fig 4D). This discrepancy in duplication may be caused, at least in part, by SRS misclassifying insertions as duplications in highly repetitive regions of the genome. For duplications, 39% (370/945) overlap SINE/Alu elements, indicating that a significant portion occurs in repetitive regions. Since SINE/Alu elements are highly similar and widespread, SRS reads may misalign or collapse duplications, leading to incorrect structural variant calls. This overlap suggests that mapping challenges in repetitive regions contribute to discrepancies in duplication detection.

### LRS Detects Longer Inversions and Resolves SRS Misclassifications

**Figure 5.**
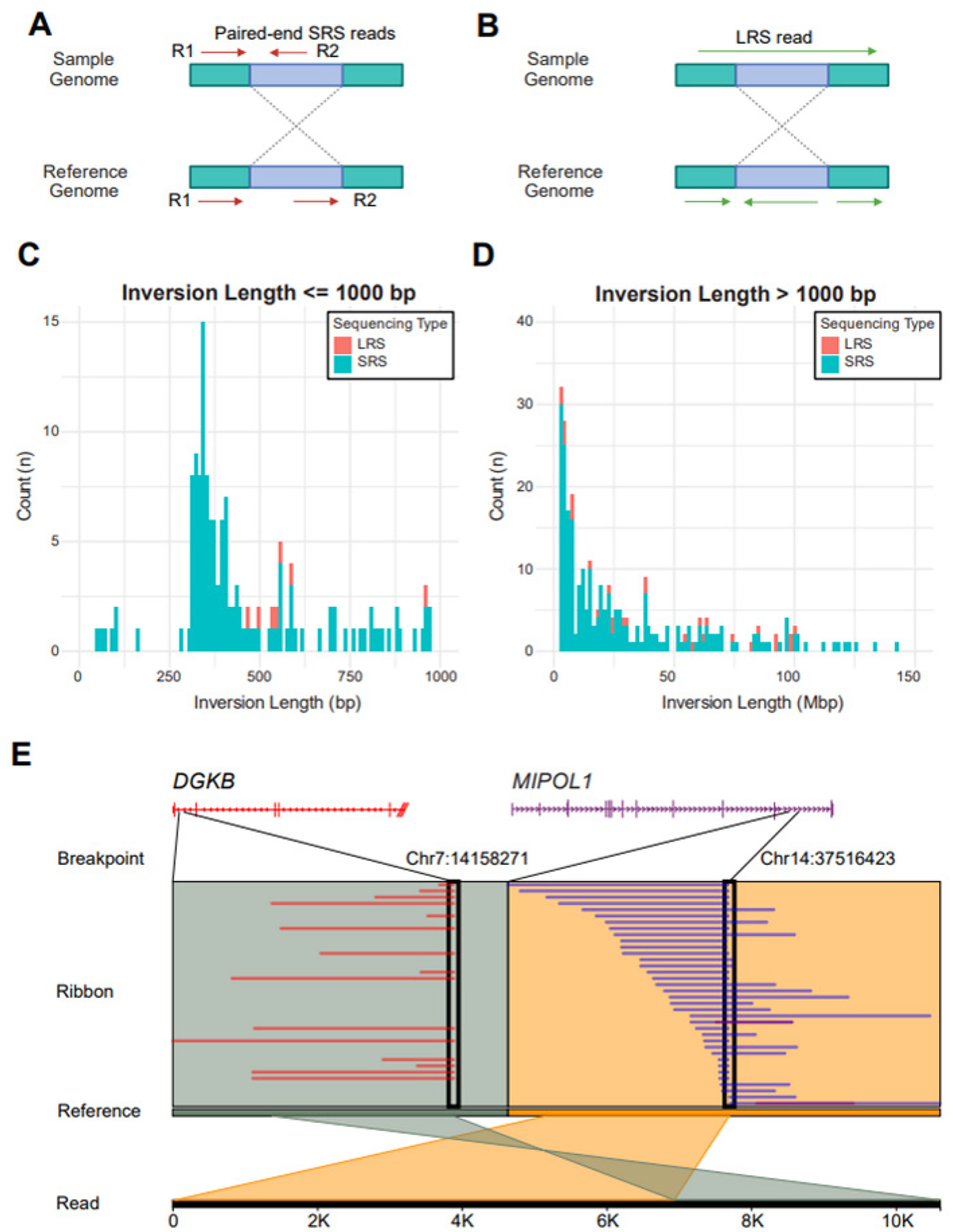
SRS detects many more inversions than LRS. **A-B.** Approaches for inversion identification by SRS and LRS. **C-D.** Stacked histograms displaying the number of inversions detected by LRS (red) and SRS (blue) for inversions ≤ 1000 bp (LRS: n=7; SRS: n=136, **C**) and inversions > 1000 bp (LRS: n=88; SRS: n=712; **D**). The x-axis represents inversion length, and the y-axis indicates the count (n) of inversions. **E.** The Ribbon plot illustrated a structural rearrangement between *DGKB* on chromosome 7 (Chr7:14158271) and *MIPOL1* on chromosome 14 (Chr14:37516423). The top panel displays gene structures (*DGKB* in red, *MIPOL1* in purple), with breakpoints marked by black rectangles. The Ribbon panel shows supporting split-read alignments, where red and purple segments indicate read mappings to *DGKB* and *MIPOL1*, respectively. The Reference panel provides a genomic alignment reference. The Read panel at the bottom visualizes mapped sequencing reads, with colored ribbons connecting homologous sequences at the breakpoints, highlighting the inversion-mediated fusion event.

SRS calls an inversion when the paired reads are oriented in the same direction when aligned to the reference genome (Fig 5A). Normally, the two reads of an SRS pair should be in opposite directions in a converging form. In contrast, LRS calls inversions when a segment of a single long read is mapped in the opposite direction on the reference genome relative to the remaining portion of the read (Fig 5B). Similar to what we have observed in duplications, we found SRS detects far more inversions than LRS (Fig 5C–D). Only SRS detected short inversions of less than 450bp, whereas both methods captured some longer inversions. RepeatMasker results show that 68% (584/855) of inversions overlap SINE/Alu elements, which are highly repetitive and prone to misalignment in SRS. Due to their high sequence similarity, SRS reads often fail to map uniquely, leading to misoriented alignments and incorrect inversion calls. This high overlap strongly suggests that many of the discrepancies in inversion detection are due to SRS mapping challenges in repetitive regions. A known *MIPOL1-DGKB* gene fusion in LNCaP mediated by inversion was also identified in LRS data and visualized using Ribbon [13] (Fig. 5E). This finding further demonstrates the capability of LRS to detect complex genomic rearrangements.

## Discussion

Our study demonstrated substantial differences in SV detection between LRS and SRS. These differences stem from variations in alignment strategies, SV calling methodologies, and the ability to detect different SV types. Most importantly, SRS is completely unable to detect insertions, which is an intrinsic limitation due to its short-read nature. Rajaby, R. et al. developed INSurVeyor [14], a tool specifically designed for calling insertions. By integrating de novo assembly strategies, it enhanced sensitivity and specificity in detecting insertions using SRS. However, LRS still holds a clear advantage in identifying insertions composed of low-complexity sequences. The repetitive nature of these sequences, along with technical challenges in accurate read mapping, makes their detection particularly difficult with SRS. Further, tandem duplications and insertions may exhibit similar mapping patterns in SRS, both appearing as discordant read pairs with increased local coverage, making them difficult to distinguish. Second, SRS does not do well at repetitive and low-complexity genomic regions, frequently causing misalignment errors and resulting in incorrect SV calls. SRS also has difficulties in detecting precise breakpoints of SVs, being consistent with previous reports [5, 7]. Like-wise, overlapping or nested SVs present a significant challenge, as short reads lack the resolution to distinguish closely spaced rearrangements, often leading to incomplete or erroneous SV reconstructions.

By contrast, LRS provides superior resolution for complex SVs and long insertions, particularly superior to SRS in repetitive regions. LRS will be extremely helpful to catalog insertions and short deletions, a majority of which have been missed or misclassified by SRS. LRS significantly improves SV detection in terms of both reliability and resolution. Moreover, long reads can span SV breakpoints with high-confidence alignments, reducing ambiguity and enhancing breakpoint resolution. Furthermore, studies have shown that LRS enables the phasing of junctions with nearby somatic and germline variants, offering a more precise resolution of complex SV haplotypes [15]. This capability provides deeper insights into SV mechanisms, functional consequences, and potential clinical relevance. Moreover, LRS demonstrates higher sensitivity in detecting small somatic SVs (≤10 kb), which are often underreported by traditional SRS approaches [8].

However, LRS could not detect deletions that are longer than its average read length, usually 10kb, due to its single-read nature. Therefore, integrating both technologies will enhance cancer genomic characterization, improving mutation annotation and clinical translation. Future studies should focus on leveraging both approaches to establish a more comprehensive and accurate SV landscape for improved diagnostics and therapeutic strategies. For example, the integration of LRS with advanced technologies like single-cell template strand sequencing (Strand-seq), a short-read sequencing-based strategy that preserves DNA strand directionality, has greatly improved the resolution of structural variation in the human genome. This approach enables the precise detection of heterozygous and homozygous inversions while enhancing the identification of full-length mobile element insertions (MEIs) [16]. By enabling a systematic investigation of MEI origins, distribution, and mobilization mechanisms, LRS offers deeper insights into complex genomic regions, including transductions [16].

In practice, Illumina is more cost-effective for large-scale, high-coverage studies with fragmented but accurate assemblies, while Nanopore is advantageous for projects requiring real-time analysis, complete metagenome-assembled genomes, and species-level resolution, albeit at a higher sequencing and computational cost [17]. Nanopore LRS faces challenges with degraded DNA, FFPE samples, and cfDNA, which can cause reduced throughput and flow cell pore blockage due to fragmentation. While Illumina SRS tolerates some DNA fragmentation, it still suffers from GC bias and uneven coverage. In contrast, Nanopore sequencing depends on long DNA fragments, making it more sensitive to degradation. Proper DNA extraction, repair, and quality control protocols are essential to mitigate these issues and improve sequencing results.

## Materials and Methods

### Oxford Nanopore Long-Read Sequencing

The Nanopore LRS DNA library was prepared as previously described [18]. Briefly, the genomic DNA of LNCaP was extracted using Quick-DNA Miniprep Plus Kit (Zymo, D4068) and fragmented to average size at 8 kb with g-TUBE^™^ (Covaris, 520079). The DNA libraries were prepared using the Ligation Sequencing Kit (ON SQK-LSK110) per the manufacturer’s protocol. The sequencing was performed on an Oxford Nanopore PromethION 2 Solo sequencer with R10.4.1 flow cells.

### Pipeline and Variant Calling

Base-calling of LRS data was performed with Dorado (version 0.8.1) using pod5 files as input to convert to fastq files. SRS data of LNCaP (SRR1977632) was downloaded from Gene Expression Omnibus. Quality control was performed on the FASTQ files using MultiQC (version 1.25.2)[19]. SRS and LRS data were aligned to the GRCh38.p14 (hg38) reference genome using BWA (v0.7.17-r1188) for SRS and Minimap2 (v2.26-r1175) with default parameters and the model dna_r10.4.1_e8.2_400bps_sup@v4.3.0 for LRS. Samtools (version 1.17) was used to convert SAM files to BAM format and generate sequencing statistics. Structural variants (SVs) were identified using Delly [11] (version 1.3.1) for SRS and the Delly LR module for LRS, with results stored in VCF files. Delly integrates paired-end mapping and split-read analysis to identify balanced and unbalanced SVs with single-nucleotide resolution. It achieves high sensitivity and specificity across a wide SV size range and supports multiple sequencing libraries with varying insert sizes. SVs shorter than 50 bp and those located on random chromosomes were labeled as “LowQual” and excluded from downstream analysis using BCFtools (version 1.21). The final SV datasets were reformatted using the query function in BCFtools for further comparison (Fig 1).

### SV Comparison Between SRS and LRS

To compare SVs of the same type between SRS and LRS, we used the GenomicRanges (version 1.50.2) and IRanges (version 2.32.0) packages [20] in R to match breakpoints within a 50 bp distance threshold. Additionally, SVs were further validated using the merge function in SURVIVOR [21] and manually inspected in IGV (version 2.16.2) [22] to confirm read alignments.

### Analysis of Repetitive Patterns in Duplications and Inversions Identified by SRS

To further explore the repetitive nature of duplications and inversions detected by SRS, we extracted the corresponding sequences from the reference genome using the samtools faidx function, based on their start and end coordinates. These sequences were saved in a .fa file and analyzed with RepeatMasker (version 4.1.7-p1) with default mode against the Dfam database [23] to identify interspersed repeats and low-complexity DNA regions. The resulting annotation table was filtered with a cutoff of Smith-Waterman (SW) score >1000 (a measure of sequence alignment quality) and %divergence (%div) < 10% (indicating minimal sequence divergence), to focus on duplications and inversions likely containing repetitive elements.

## Figures and Tables

**Table 1.** Summary of sequencing information in two different datasets

Dataset	Sequencing Technology	Average Read Length (bp)	Reads Yield	Coverage (X)
NP-0056	ONT	3,736	58,245,198	32
SRR1977632	Illumina	90	2,517,372,955	63

**Table 2.** Summary of detected SVs across two different datasets

	Deletion	Duplication	Inversion	Insertion
SRS-WGS of LNCaP	3,557	945	855	0
LRS-WGS of LNCaP	9,072	99	96	16,114
Overlapping	2,178	28	49	0

## References

[1] Tomlins SA, Rhodes DR, Perner S, Dhanase-karan SM, Mehra R, Sun XW, Varambally S, Cao X, Tchinda J, Kuefer R : Recurrent fusion of TMPRSS2 and ETS transcription factor genes in prostate cancer. Science 2005, 310(5748):644–648. 16254181 10.1126/science.1117679

[2] Quigley DA, Dang HX, Zhao SG, Lloyd P, Aggarwal R, Alumkal JJ, Foye A, Kothari V, Perry MD, Bailey AM, : Genomic Hallmarks and Structural Variation in Metastatic Prostate Cancer. Cell 2018, 174(3):758–769 e759. doi:10.1016/j.cell.2018.06.039. 30033370 PMC6425931

[3] Sienkiewicz K, Yang C, Paschal BM, Ratan A: Genomic analyses of the metastasis-derived prostate cancer cell lines LNCaP, VCaP, and PC3-AR. Prostate 2022, 82(4):442–451. doi:10.1002/pros.24290. 34951700 PMC8792310

[4] Seim I, Jeffery PL, Thomas PB, Nelson CC, Chopin LK: Whole-Genome Sequence of the Metastatic PC3 and LNCaP Human Prostate Cancer Cell Lines. G3 (Bethesda) 2017, 7(6):1731–1741. doi:10.1534/g3.117.039909. 28413162 PMC5473753

[5] Choo ZN, Behr JM, Deshpande A, Hadi K, Yao X, Tian H, Takai K, Zakusilo G, Rosiene J, Da Cruz Paula A : Most large structural variants in cancer genomes can be detected without long reads. Nat Genet 2023, 55(12):2139–2148. doi:10.1038/s41588-023-01540-6:37945902 PMC10703688

[6] Lucas Lledo JI, Caceres M: On the power and the systematic biases of the detection of chromosomal inversions by paired-end genome sequencing. PLoS One 2013, 8(4):e61292. doi:10.1371/journal.pone.0061292. 23637806 PMC3634047

[7] Mahmoud M, Gobet N, Cruz-Davalos DI, Mounier N, Dessimoz C, Sedlazeck FJ: Structural variant calling: the long and the short of it. Genome Biol 2019, 20(1):246. doi:10.1186/s13059-019-1828-7. 31747936 PMC6868818

[8] Sedlazeck FJ, Rescheneder P, Smolka M, Fang H, Nattestad M, von Haeseler A, Schatz MC: Accurate detection of complex structural variations using single-molecule sequencing. Nat Methods 2018, 15(6):461–468. doi:10.1038/s41592-018-0001-7. 29713083 PMC5990442

[9] Kumar A, Haggblom MM, Kerkhof LJ: A Step-by-Step Guide to Sequencing and Assembly of Complete Bacterial Genomes Using the Oxford Nanopore MinION. Methods Mol Biol 2025, 2866:31–43. doi:10.1007/978-1-0716-4192-7_2:39546195

[10] Li H: Minimap2: pairwise alignment for nucleotide sequences. Bioinformatics 2018, 34(18):3094–3100. doi:10.1093/bioinformatics/bty191. 29750242 PMC6137996

[11] Rausch T, Zichner T, Schlattl A, Stutz AM, Benes V, Korbel JO: DELLY: structural variant discovery by integrated paired-end and split-read analysis. Bioinformatics 2012, 28(18):i333–i339. doi:10.1093/bioinformatics/bts378. 22962449 PMC3436805

[12] Danecek P, Bonfield JK, Liddle J, Marshall J, Ohan V, Pollard MO, Whitwham A, Keane T, McCarthy SA, Davies RM : Twelve years of SAMtools and BCFtools. Gigascience 2021, 10(2). doi:10.1093/gigascience/giab008. PMC7931819 33590861

[13] Nattestad M, Aboukhalil R, Chin CS, Schatz MC: Ribbon: intuitive visualization for complex genomic variation. Bioinformatics 2021, 37(3):413–415. doi:10.1093/bioinformatics/btaa680. 32766814 PMC8058763

[14] Rajaby R, Liu DX, Au CH, Cheung YT, Lau AYT, Yang QY, Sung WK: INSurVeyor: improving insertion calling from short read sequencing data. Nat Commun 2023, 14(1):3243. doi:10.1038/s41467-023-38870-2. 37277343 PMC10241795

[15] Setton J, Hadi K, Choo ZN, Kuchin KS, Tian H, Da Cruz Paula A, Rosiene J, Selenica P, Behr J, Yao X, : Long-molecule scars of backup DNA repair in BRCA1- and BRCA2-deficient cancers. Nature 2023, 621(7977):129–137. doi:10.1038/s41586-023-06461-2:37587346 PMC10482687

[16] Ebert P, Audano PA, Zhu Q, Rodriguez-Martin B, Porubsky D, Bonder MJ, Sulovari A, Ebler J, Zhou W, Serra Mari R : Haplotype-resolved diverse human genomes and integrated analysis of structural variation. Science 2021, 372(6537). doi:10.1126/science.abf7117. PMC8026704 33632895

[17] Xia Y, Li X, Wu Z, Nie C, Cheng Z, Sun Y, Liu L, Zhang T: Strategies and tools in illumina and nanopore-integrated metagenomic analysis of microbiome data. Imeta 2023, 2(1):e72. doi:10.1002/imt2.72. 38868337 PMC10989838

[18] Lu X, Keo V, Cheng I, Xie W, Gritsina G, Wang J, Jin Q, Jin P, Yue F, Sanda MG, : Epigenetic remodeling and 3D chromatin reorganization governed by NKX2–1 drive neuroendocrine prostate cancer. bioRxiv 2024. doi:10.1101/2024.12.04.626816.

[19] Ewels P, Magnusson M, Lundin S, Kaller M: MultiQC: summarize analysis results for multiple tools and samples in a single report. Bioinformatics 2016, 32(19):3047–3048. doi:10.1093/bioinformatics/btw354. 27312411 PMC5039924

[20] Lawrence M, Huber W, Pages H, Aboyoun P, Carlson M, Gentleman R, Morgan MT, Carey VJ: Software for computing and annotating genomic ranges. PLoS Comput Biol 2013, 9(8):e1003118. doi:10.1371/journal.pcbi.1003118. 23950696 PMC3738458

[21] Jeffares DC, Jolly C, Hoti M, Speed D, Shaw L, Rallis C, Balloux F, Dessimoz C, Bahler J, Sedlazeck FJ: Transient structural variations have strong effects on quantitative traits and reproductive isolation in fission yeast. Nat Commun 2017, 8:14061. doi:10.1038/ncomms14061. 28117401 PMC5286201

[22] Robinson JT, Thorvaldsdottir H, Wenger AM, Zehir A, Mesirov JP: Variant Review with the Integrative Genomics Viewer. Cancer Res 2017, 77(21):e31–e34. doi:10.1158/0008-5472.CAN-17-0337. 29092934 PMC5678989

[23] Storer J, Hubley R, Rosen J, Wheeler TJ, Smit AF: The Dfam community resource of transposable element families, sequence models, and genome annotations. Mob DNA 2021, 12(1):2. doi:10.1186/s13100-020-00230-y. 33436076 PMC7805219

